# Platform science and the fragmentation of global scientific cooperation

**DOI:** 10.1093/pnasnexus/pgag140

**Published:** 2026-06-02

**Authors:** Vaughan C Turekian

**Affiliations:** Executive Director, International Networks, Cooperation and Security, US National Academies of Sciences, Engineering and Medicine, 500 Fifth Street, NW, Washington, DC 20001, USA

The conduct of science is entering a new phase, shaped by the convergence of AI, high-performance computing, and increasingly automated experimental systems. While discovery continues to rely on theory and experiment, it is increasingly augmented by integrated platforms that link data, models, computing, and instrumentation at scale. In fields such as materials science and structural biology, these approaches are already accelerating discovery—exemplified by tools such as AlphaFold in protein structure prediction and AI-driven materials discovery—by connecting simulation, data, and experiment in new ways. The US Department of Energy's Genesis Mission exemplifies this broader shift: it aims to build a unified AI-enabled scientific infrastructure—integrating computing, data, and experimental systems—to accelerate discovery across domains from energy materials to biological systems (1). As such platform-based approaches expand, they are likely to reshape the conditions for international scientific collaboration, as access to data, models, and computing becomes increasingly governed by divergent and durable policy regimes. The simultaneous rise of AI-enabled research platforms and the fragmentation of global technology governance together demand a new model of science and technology diplomacy—one oriented not toward facilitating exchange but toward structuring participation across systems that are unlikely to converge.

**Figure:**
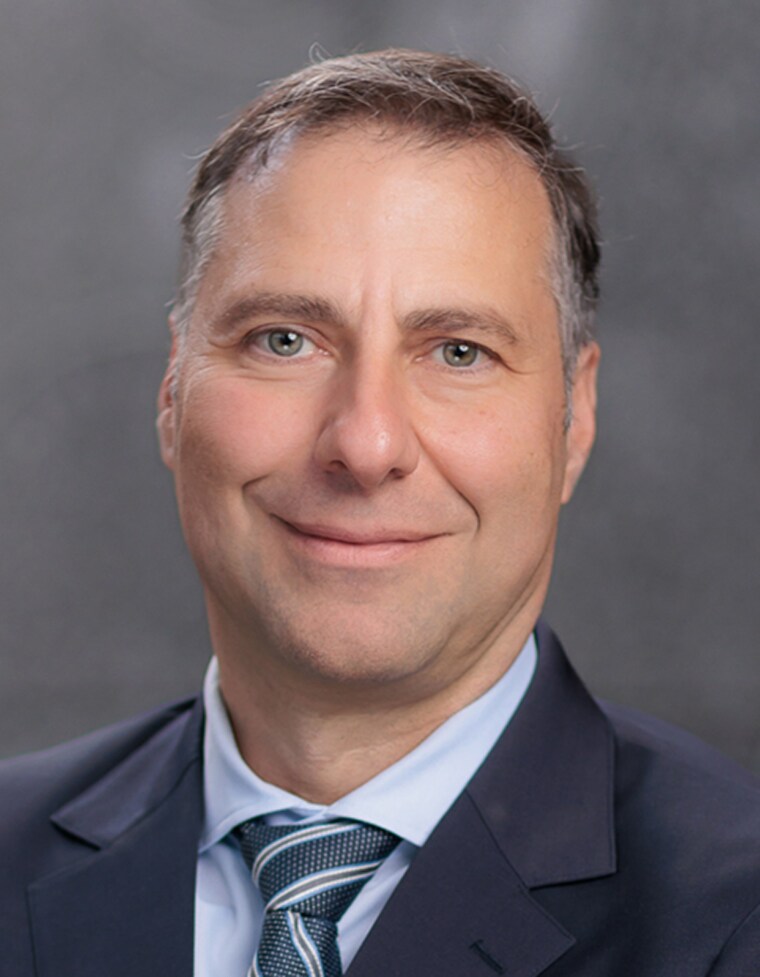
Vaughan C. Turekian

These trends extend beyond academic science to the broader research and development ecosystem, including industrial R&D in areas such as pharmaceuticals, where large-scale data, models, and computational infrastructure are central to innovation. Together, they point not simply to a change in tools but to a reorganization of the institutional and collaborative foundations of modern science. In this emerging context, large-scale, AI-enabled systems are giving rise to what might be called technopoles for science: integrated ecosystems that combine infrastructure, data resources, and institutional capacity to enable research at scale. The concept of technopoles was previously developed in the context of geopolitical competition over science and technology, where nations leveraging science and technology as strategic assets increasingly define power in the multipolar world (2). Here, the concept takes on an additional dimension: within nations and across allied networks, the concentration of AI-enabled research infrastructure creates nodes of scientific capability that function analogously—structuring access, shaping participation, and defining the geography of discovery itself. These systems constitute an emerging geography of scientific capability, in which access to advanced platforms increasingly determines participation in discovery. As capability becomes more concentrated in these environments, access to infrastructure is increasingly likely to become a primary mechanism through which both scientific opportunity and collaboration are structured.

This transformation is unfolding alongside a global landscape characterized by divergence in the governance of AI-enabled research systems. Governments are adopting distinct and persistent approaches to issues such as data use, model access, and technology control, reflecting differing regulatory traditions, societal priorities, and approaches to the governance of technology. The European Union's AI Act, with its risk-based regulatory tiers and data governance requirements, diverges substantially from the United States’ more sectoral and incentive-oriented approach; both differ markedly from China's model, which combines state-directed data access with algorithmic governance designed around national strategic priorities (3, 4). Overlaid on these AI-specific frameworks are data localization requirements, export controls on advanced semiconductors and AI models, and expanding research security policies that increasingly govern institutional-level collaboration. These differences are unlikely to converge in the near term. As a result, the same platforms that enable scientific progress are embedded within governance frameworks that shape how they can be accessed, integrated, and used across borders. These dynamics are further reinforced by the growing prominence of research security policies, which increasingly shape collaboration at the institutional level.

Against this backdrop, the implications for international scientific collaboration become more apparent. Historically, large-scale scientific endeavors—such as the Large Hadron Collider or ITER—benefited from a relatively high degree of international openness, supported by shared norms and governance frameworks. AI-enabled science introduces new constraints. Because underlying models can be dual-use and data may carry strategic or sensitive characteristics, participation in emerging platforms is increasingly conditioned by trust and compatibility across governance regimes. In parallel, research security policies across many countries are strengthening protections around sensitive technologies, data, and intellectual property. While these efforts respond to legitimate concerns, they also introduce additional layers of divergence in how collaboration is structured. The result is a shift toward more explicitly governed forms of international engagement, in which access to infrastructure and data is negotiated within overlapping policy frameworks.

The interaction between AI governance and research security is particularly significant for platform-based science. Integrated systems that combine data, models, and computing resources concentrate both opportunity and sensitivity. Access to these platforms therefore becomes a focal point where scientific, regulatory, and security considerations converge. Participation in advanced scientific ecosystems is increasingly shaped—and in some cases constrained—by overlapping and sometimes incompatible governance regimes rather than a single set of shared norms. In domains involving individual-level data, fragmentation extends beyond access to differences in how data can be used within AI systems. Across jurisdictions, variations in human-subjects oversight, data protection frameworks, and emerging AI governance regimes create divergent constraints on secondary use, model training, and the inference of sensitive attributes, such that the same data may be accessible but not equivalently usable across platforms (5). At its core, this transformation is redefining how international scientific collaboration is structured, who can participate, and under what conditions. Taken together, these dynamics suggest that sustaining international scientific cooperation in the era of platform-based science will depend on the deliberate development of mechanisms that can operate across divergent governance systems.

In this evolving environment, international scientific cooperation is best understood not as a binary choice between openness and restriction, but as a set of differentiated arrangements. Collaboration increasingly takes multiple forms, ranging from deep integration among closely aligned partners to more modular or project-based engagement across broader networks. At the same time, some domains of research—particularly in lower-risk areas of basic science—may continue to support relatively open exchange. The result is a more stratified landscape of scientific cooperation, shaped by both technological architecture and governance compatibility.

Science and technology diplomacy, therefore, takes on a more operational role within this environment. It becomes a primary mechanism through which international scientific cooperation can be structured and sustained across divergent governance systems. As previously argued, science diplomacy must evolve from its post-Cold War aspirational model of open exchange toward a more transactional and explicitly structured form of engagement (6). This marks a shift from science diplomacy as a facilitator of exchange to a mechanism for structuring participation in shared scientific systems. This includes developing interoperable approaches to data and research practices, aligning expectations around access to models and infrastructure, and creating mechanisms that enable participation without requiring full regulatory convergence. Emerging initiatives—such as the Quad's Critical and Emerging Technologies Working Group and bilateral science and technology agreements that increasingly address data sharing and model access—suggest the outlines of what more structured platform-era diplomacy might look like. In this sense, science diplomacy becomes a means of managing difference, enabling collaboration across systems that are not aligned.

These developments raise important questions for science policy. As countries invest in AI-enabled scientific infrastructure, there is a need to consider how such systems can support both national research priorities and international collaboration. This includes developing clearer frameworks for participation in large-scale platforms, such as how access is determined and under what conditions. It also requires attention to the institutional arrangements—such as partnership models and resource contributions—that shape participation in shared infrastructure. For the United States, which is investing heavily in AI-enabled scientific infrastructure, this creates an opportunity to help shape the design of these mechanisms—both through the development of domestic platforms and through international engagement that defines how collaboration can occur across differing governance systems. At the same time, policymakers will need to identify domains of research where broader openness continues to advance both scientific and societal interests.

There are also risks associated with this evolving landscape. If access to advanced platforms becomes too narrowly defined, segments of the international scientific community—particularly those in resource-constrained settings—may face increasing barriers to participation in emerging areas of discovery. Such outcomes could limit the diversity of scientific perspectives and reduce opportunities for global knowledge exchange. Addressing these challenges will require deliberate efforts to create pathways for broader engagement, particularly in areas where risks are lower and the benefits of inclusion are high.

At the same time, the emergence of more structured systems of collaboration does not imply a retreat from international science. Rather, it reflects a transition toward new forms of scientific organization, in which cooperation is more explicitly shaped by infrastructure, governance, and institutional design. Understanding how to design these systems in ways that sustain both scientific progress and meaningful international collaboration will be an important task for the coming decade.

The future of scientific progress will depend not only on advances in AI and computing but also on how effectively scientific systems are organized and connected. As AI becomes more deeply embedded in the infrastructure of discovery, the ability to sustain collaboration across differing governance contexts will become increasingly important. The challenge is not to eliminate fragmentation—that ambition is neither achievable nor, in all respects, desirable. It is to ensure that the architecture of platform-based science is designed from the outset with international participation in mind: that access mechanisms, data standards, and governance frameworks are built to enable collaboration across systems that will not converge. This requires deliberate choices by scientists, institutions, and governments now, while these platforms are still being designed. The decisions made in the next 5 years about who can access AI-enabled scientific infrastructure, and under what conditions, will shape the geography of global discovery for a generation.
